# The Use of Barbed Sutures in Total Hip Arthroplasty: A Systematic Review on Clinical–Surgical Outcomes, Costs, and Complications

**DOI:** 10.3390/healthcare12111063

**Published:** 2024-05-23

**Authors:** Antonio Russo, Michele Centola, Alberto Nicodemo, Alessandro Massè

**Affiliations:** 1 Humanitas Torino, Via Cellini 5, 10126 Turin, Italy; michcentola@gmail.com (M.C.); nicodemo.a@libero.it (A.N.); 2 Department of Surgical Sciences, University of Turin, Corso Dogliotti 24, 10126 Turin, Italy; alessandro.masse@unito.it

**Keywords:** barbed sutures, barbed technology, total hip arthroplasty, THA, TJR, hip replacement

## Abstract

Purpose: Total hip arthroplasty is among the most successful procedures in orthopaedic surgery. As the total number of total hip arthroplasties is constantly rising and it is expected to further increase, efforts oriented to optimise surgical pathways are investigated, aiming to reduce complications and diminish costs. The wound suturing phase is one of the steps that may be addressed. Barbed sutures have proved to reduce surgical times and enhance suture stability, then reducing wound-related complications in many surgical fields. The evidence on the use of this technology in total hip arthroplasty is still sparse, and its effect on patient outcomes and costs must still be clarified. Methods: A systematic search of studies published from 1 January 2000 to 1 March 2023 was performed. Two authors independently reviewed the literature available in eight electronic databases to identify papers eligible for inclusion. Results: A total of nine studies investigating 6959 procedures on 6959 patients were included in the final analysis. Five studies were randomised controlled trials, and the overall quality of studies ranged from moderate to high. The mean age of patients ranged from 43.8 to 70 years. BMI ranged from 25 to 31.9 kg/m^2^. The mean follow-up of studies ranged from 3 to 6 months. Conclusions: Evidence included in the systematic review suggested that the use of barbed sutures is associated with lower suturing times, complication rates, and overall costs when compared to the use of traditional suturing techniques. Level of evidence: II, systematic review of level I and II studies.

## 1. Introduction

As total hip arthroplasty (THA) is one of the most successful and reproducible procedures in orthopaedic surgery, its total number has consistently risen in recent years, and it is expected to continue to rise in the future [1,2,3]. Beyond the optimal surgical technique and implant positioning, appropriate soft tissues management is fundamental to avoid complications that may ultimately jeopardise outcomes of THA [4,5]. Although the importance of wound suturing is often overlooked, it does constitute a variable that could be optimised to improve clinical outcomes and patient satisfaction [6,7]. However, traditional suturing techniques that include continuous or interrupted sutures have not changed over the last decades. More recently, novel suture techniques using knotless barbed sutures have been introduced as alternatives to traditional methods [8,9]. Theoretically, closure with barbed sutures should guarantee water-tight wounds and provide some advantages over traditional sutures, such as reduced closure time, wound infections, hematoma, dehiscence, and total healthcare costs [10,11,12,13]. The use of barbed sutures has already been extensively investigated in gynaecologic, urologic, laparoscopic, and plastic surgery [14,15,16]. In the field of orthopaedic surgery, this technology has been assessed for flexor tendon repair, where it was demonstrated to achieve similar or even greater strength when compared to traditional techniques [17,18]. Within total joint arthroplasty (TJA), barbed sutures have been studied for closure of arthrotomy, fascial, and subcutaneous layers. Most of these studies are focused exclusively on a cohort of patients who underwent total knee arthroplasty (TKA) and mixed TKA-THA cohorts, but there is a general paucity of papers examining barbed sutures in the setting of THA [10,19,20]. A meta-analysis of randomised controlled trials (RCT) by Han et al. [21] demonstrated lower costs and operative times and similar functional outcomes when barbed sutures were used compared to traditional sutures. However, of the six papers included in this study, only two included a mixed cohort of patients who underwent THA or TKA, and functional outcomes were available only for TKA patients. More recently, Sun et al. [22] conducted a meta-analysis of RCTs and compared outcomes of patients who had TKA and were sutured with barbed sutures to patients who had traditional suturing, showing a lower incidence of peri-incisional ecchymosis and shorter total wound closure time. However, because of differences in anatomy, biomechanics, and the resulting forces on the soft tissue envelope between hip and knee joints, the results of studies conducted on patients with TKA may not apply to patients who had THA. To date, there is no systematic analysis of available evidence focused specifically on the use of barbed sutures in THA. Considering these points, we designed this systematic review of the literature aiming to summarise the evidence available on the use of barbed sutures for wound closure in THA and to analyse clinical–surgical outcomes provided by this technology and costs.

## 2. Methods

**Figure 1:**
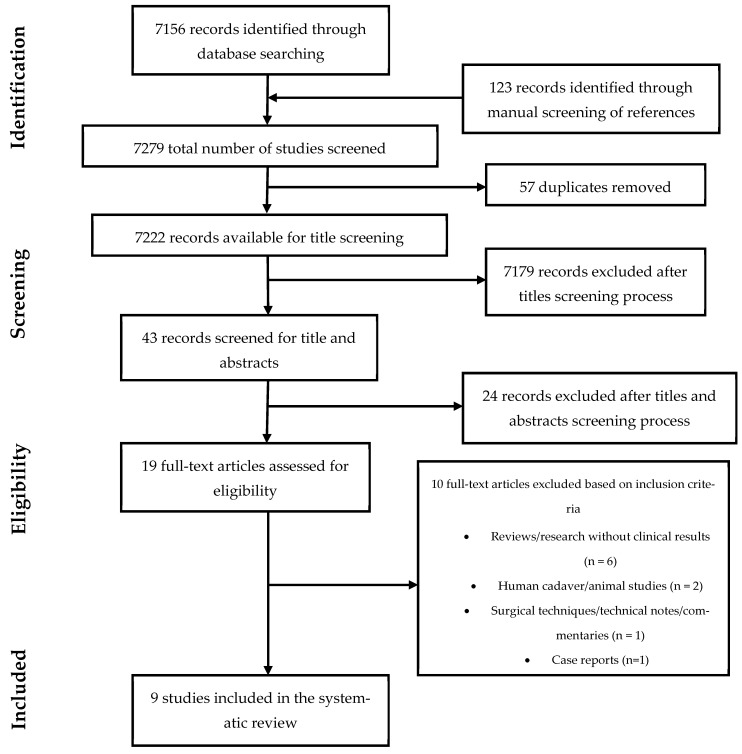
PRISMA flowchart for studies selection.

The present systematic review was conducted according to the *Cochrane Handbook of Systematic Reviews of Interventions* [23]. The process of study selection was reported using the flowchart proposed by the Preferred Reporting Items for Systematic Reviews and Meta-Analyses (PRISMA) [24] (Figure 1). In the period ranging from 1 January 2000 to 1 March 2023, the keywords “hip arthroplasty”, “hip replacement”, “barbed suture”, “joint replacement”, “hip arthritis”, and “osteoarthritis” were used for searching the Cochrane Central Register of Controlled Trials (CENTRAL), Scopus, ScienceDirect, MEDLINE/PubMed, Embase, the Science Citation Index Expanded from Web of Science, Lilacs, and CINAHL. Original research focused on patients who underwent THA and wound closure with barbed sutures was considered suitable for the final analysis, whereas case reports, surgical techniques, abstracts, editorial commentaries, and pre-clinical studies were excluded. A total of 7279 studies were initially identified for screening. Two reviewers (A.R. and M.C.) screened the list of papers provided by the primary search, aiming to select titles and abstracts relevant for the purpose of the review and to detect duplicates. Any possible disagreement between the two reviewers was solved by including in the selection process a third senior reviewer (A.N.). After exclusion criteria were applied, full-text assessment of papers that were considered potentially eligible was completed. After the application of exclusion criteria, nine studies were eventually included in the systematic review (see Figure 1). The patient, intervention, comparison, outcomes, study design (PICOS) was used to assess and answer questions according to the PRISMA checklist: patient (P), patients who underwent THA; intervention (I), patients who underwent THA and sutures with barbed technology; comparison (C), patients who underwent THA and sutured with traditional technology; outcomes (O), clinical-surgical outcomes, operative times, blood loss, complications, and costs. 

### 2.1. Quality of Evidence

The adjusted Oxford Centre for Evidence-Based Medicine 2011 Levels of Evidence [25] was used to assess the level of evidence and the quality of studies was classified through the grading of recommendations, assessment, development, and evaluations (GRADE) [26] system. Of the studies included, five were level of evidence I, two had a level of evidence II, and two had a level of evidence III, and the overall quality ranged from moderate to high (Table 1). The risk of bias was defined using the Methodological Index for Non-Randomised Studies (MINORS) [27]. MINORS scores of each study are provided within Table 2. According to the MINORS criteria, there was a high risk of bias in one of the included studies and moderate risk in the remaining eight studies.

### 2.2. Endpoints and Statistical Analysis

Complication rates, blood loss, closure time, and costs were considered as the primary endpoints of this systematic review. When studies were conducted on mixed hip and knee cohorts, analysis was focused specifically on data retrieved from patients who underwent THA. Categorical variables were presented as a number of events or percentage. Continuous variables were expressed as means weighted on the number of procedures. IBM SPSS Statistics version 26.0 (IBM Corp., Armonk, NY, USA) was used to conduct the statistical analysis. The general characteristics of the studies were extracted and presented within Table 1. Data related to the primary endpoints of the review were tabulated in Table 3.

## 3. Results

A total of 6959 procedures on 6959 patients were included for analysis in this review. The mean age of the patients of the included studies ranged from 43.8 to 70 years. BMI ranged from 25 to 31.9 kg/m^2^. The mean follow-up of studies ranged from 3 to 6 months. Detailed demographic data are displayed in Table 1.

### 3.1. Blood Loss

Only the study of Knapper et al. [28], which compared the use of barbed sutures to staples for the closure of the skin, assessed the variable blood loss. They found significant lower perioperative blood loss (mean 432 mL, *p* = 0.006) and days elapsed before the wound was completely dry (0 vs. 1, *p* < 0.0001) in the barbed-suture group.

### 3.2. Closure Time

Of the studies included, six evaluated closure time. Ting et al. [20] demonstrated a significant reduction in time for closure using barbed sutures in all three layers (*p* = 0.0218). Li et al. [29] showed a mean reduction of 4.21 min (*p* < 0.001) for fascia and 6.25 min (*p* < 0.001) for the overall closure time. Conversely, Serrano Chinchilla et al. [30] reported that global closing time was shorter in the barbed-suture group (5’59”) compared to controls (7′1″), (*p* < 0.04), mean 37″ difference in subcutaneous closure (*p* = 0.048) but no difference in the fascial layer. Sunderam et al. [11] detected a significant reduction in arthrotomy closing time (3 vs. 8 min, *p* < 0.001) when using barbed sutures, which resulted in a significant reduction in total closing time (*p* = 0.02). Wang et al. [31] used barbed sutures for fascial and subcutaneous layers and compared different approaches for skin closure, reporting mean global suturing time ranging from 7 to 13 min. Smith et al. [32], analysing their mixed hip and knee cohort, showed a significantly lower closing time in all layers (*p* < 0.001).

### 3.3. Costs

The larger study that included assessing costs was by Sutton et al. [33]. This paper analysed data from 5958 patients and concluded that patients who received wound closure with barbed sutures had a significantly lower mean hospital length-of-stay (LOS) (2.5 vs. 2.8 days, *p* = 0.002), mean operating room time (183 vs. 190 min, *p* = 0.0235), and rate of discharge to skilled nurse facilities (SNF) (21.7 vs. 28.5%, *p* = 0.013) when compared to traditional sutures, which resulted in a non-significant lower mean total cost (USD 7038 vs. USD 18,144; *p* = 0.103). Ting et al. [20] registered a higher cost for the barbed sutures when compared to traditional ones (USD 52.75 ± 19.96 vs. 12.79 ± 1.95, *p* = 0.008); however, considering the significant lower closure time, they estimated a compound saving of USD 614.72 ± 340.13 per patient related to the use of barbed technology. Similarly, Smith et al. [32] reported higher costs for barbed sutures when considering the cost of the sutures alone but a total saving of USD 549.59 per case considering the lower operating room time and costs. Li et al. [29] reported higher costs for the barbed-suture group considering the cost of the sutures alone (Table 3).

### 3.4. Complications

Of the seven studies that conducted statistical comparisons in the wound-related complication rate, six found no significant differences between patients who received barbed compared to traditional sutures [11,20,28,29,30,32]. The study by Thacher et al. [34] reported a significantly higher rate of dehiscence in the barbed group compared to the traditional sutures group (3 vs. 0.7%, *p* = 0.04) but a lower rate of superficial wound infection (0 vs. 5%, *p* = 0.001).

## 4. Discussion

This present systematic review of the literature highlighted that the use of barbed sutures in the context of THA is associated with overall low closure time and complication rates and, consequently, lower costs for the healthcare system when compared to the use of traditional sutures. THA has become an increasingly common and successful surgical procedure in the last four decades, and it is expected to further increase in the future. Then, optimisation of this procedure and the related clinical pathway is crucial, aiming to reduce the overall cost for the healthcare system. One of the possible strategies is to increase the quality of the suturing phase [35]. In fact, even if often overlooked, an appropriate suturing technique has a primary relevance for the overall outcome of surgeries and can help prevent devastating complications [36,37]. Barbed sutures have already been proven to result in excellent outcomes in hand surgery and surgery of the knee. A recent meta-analysis by Sun et al. [22] showed that barbed sutures resulted in shorter total wound closure time (*p* < 0.001) and fewer needle puncture injuries to members of the surgical team (*p* = 0.02) in TKA, whereas there were no significant differences in blister formation (*p* = 1.0), superficial infection (*p* = 0.82), range of motion (*p* = 0.94), incisional exudate (*p* = 0.75), suture abscess (*p* = 0.26), suture breakage (*p* = 0.11), wound-related complications (*p* = 0.10), or ecchymosis (*p* = 0.08) between barbed and conventional wound closures [22]. However, outcomes from TKA cohorts should not be extended to outcomes expected in THA due to the anatomical and biomechanical differences in these districts. Globally, studies included in this analysis showed that operative times are significantly impacted using barbed technology. Although studies investigating different suturing techniques have been included, all demonstrated a reduction in suturing times, at least in the phase in which the barbed suture was used. 

All the studies highlighted higher costs of barbed sutures compared to traditional technologies [20,29,32]. Nevertheless, some studies demonstrated overall cost savings of around USD 600 per case [20,32]. The study by Sutton et al. [33], that beyond operative room costs included in the analysis were also costs related to readmission, complications, LOS, and patients discharge to non-home facilities, estimated that a mean of USD 2000 was saved per case when using barbed sutures. Complications were considered statistically comparable in patients sutured with barbed technology or traditional sutures in most of the studies. However, overall analysis showed a significantly higher rate of complications in patients who received traditional sutures (7.2 vs. 4.8%, *p* < 0.001). All these considerations suggest that barbed sutures have a potential in reducing operative times, OR costs, and wound-related complications, which ultimately have a strong impact on a patient’s quality of life and overall healthcare costs. Only one study included in this systematic review analysed blood loss as a variable, demonstrating reduced blood loss and LOS in the group of patients sutured with the barbed technology. However, this finding must be interpreted with caution, since the authors stated that the barbed closure was used only for the skin layer.

It should be highlighted that in addition to measurable variables, such as those included in this systematic review, the use of barbed sutures may result in reduced peri-incisional haematoma and oedema, eventually leading to reduced patient discomfort and accelerated rehabilitation.

It must be noted that several limitations affect this study. First, while most of the studies were classified as high-quality evidence, they were all included in this review despite that they analysed different protocols of wound suturing. In fact, in some studies, barbed sutures were used to close all of the layers (fascia, subcutaneous fat, subcuticular, and skin), while some other authors used the barbed technology only in one or some of these, using traditional sutures for the remaining layers. Moreover, some papers included were retrospective or prospective studies, but certain variables were analysed retrospectively. This methodology has inherent limitations since some variables, such as the number of complications, could have been missed or underestimated. Only a few studies claimed that surgeons performed several cases using the barbed technology to become familiar with it before starting the study, and this can have an impact on the overall outcomes of the analysis. Even though wound-related complications often appear in the first post-operative period, the short follow-up of studies can constitute one further limitation. Finally, outcomes like costs and suturing times were reported using non-homogeneous measures among studies, complicating their overall analysis.

## 5. Conclusions

The use of barbed sutures for wound closure of THA guarantees lower suturing time and wound-related complication rates when compared to the use of traditional sutures. Cost analysis revealed general higher costs for barbed sutures alone compared to traditional technologies but an overall cost saving when considering variables such as OR time and hospital LOS. In conclusion, the use of barbed sutures should be incentivised in THA since they could contribute to lower complications, OR time, LOS, and consequently, overall healthcare costs.

## Figures and Tables

**Table 1:** General characteristics of studies included. BMI, body mass index; mo, months; RCT, randomised controlled trial; w, weeks. * data related to the overall population.

First Author	Year	Country	Number of Patients	Number of Procedures	BMI	Sex	Age (Years)	Study Design	Level of Evidence	GRADE	Follow-Up
Ting	2012	USA	25	25	31.3 *	-	63.95 *	RCT	I	High	3 mo
Smith	2014	USA	16	16	31.9	7 M 9 F	58.7	RCT	I	High	-
Li	2018	China	46	46	-	32 M 14 F	43.76	RCT	I	Moderate	6 w
Sutton	2018	USA	5958	5958	-	2473 M 3485 F	65.8	Retrospective	III	Moderate	-
Knapper	2019	UK	84	84	31	29 M 55 F	70	Prospective	II	Moderate	6 mo
Thacher	2019	USA	591	591	27.9	244 M 347 F	66.3	Retrospective	III	Moderate	3 mo
Serrano Chinchilla	2020	Spain	82	82	29	40 M 42 F	66.1	RCT	I	High	1 mo
Wang	2020	China	97	97	25	-	54.2	Prospective	II	Moderate	3 mo
Sunderam	2021	USA	60	60	29.5	28 M 32 F	63.5	RCT	I	High	3 mo

**Table 2:** MINORS criteria.

First Author	Clearly Stated Aim	Inclusion of Consecutive Patients	Prospective Collection of Data	Endpoint Appropriated to the Aim of the Study	Unbiased Assessment of the Study Endpoint	Follow-Up Period Appropriate to the Aim of the Study	Loss to Follow-Up Less than 5%	Prospective Calculation of the Study Size	An Adequate Control Group	Contemporary Groups	Baseline Equivalence of Groups	Adequate Statistical Analyses	Total Points
Ting	2	2	0	2	1	2	2	0	2	2	1	2	18
Smith	2	1	2	2	1	2	0	2	2	1	1	2	18
Li	2	1	0	2	1	2	2	0	2	2	1	2	17
Sutton	2	1	0	2	1	2	0	0	2	2	2	2	16
Knapper	2	1	0	2	1	1	0	0	2	2	1	2	14
Thacher	2	1	0	2	2	2	2	0	2	2	2	2	19
Serrano Chinchilla	2	1	0	2	2	2	1	0	2	2	2	2	17
Wang	2	2	0	2	2	2	2	0	2	2	2	2	16
Sunderam	2	2	0	2	2	2	2	0	2	2	1	2	19

**Table 3:** Outcomes assessed by the studies included in the systematic review. B, barbed; PJI, periprosthetic joint infection; SD, standard deviation, T, traditional. * data related to the overall population.

First Author	Intervention	Layer Evaluated	Blood Loss (mL)	Closure Time Mean (min) ± SD	Cost ± SD	Type of Suture	N. of Sutures	Complications
Ting	B	Fascia, subcutaneous fat, subdermal	-	9.6	52.75 ± 19.96 $ per patient	Polydioxanon	2.6	1 peri-incisional erythema
	T			15.0	12.79 ± 1.95 $ per patient	Vicryl, Monocryl, Dermabond	6.5	
Smith	B	Fascia, fat, subcutaneous, subcuticular	-	16.7 *	116.9 * $ per patient	Quill, Quill monoderm	4.6 *	3 * superficial site infection
	T			26.5 *	8.0 * $ per patient	Vicryl, Monocryl	6.9 *	1 * prominent suture
Li	B	Fascia, subcutaneous fat		12	128.3 * RMB per patient	Quill, Vycril, Staples	-	2 redness, 1 exudation, 1 skin allergy
	T			18.25	497.2 * RMB per patient	Vycril, Staples		1 redness, 1 exudation, 1 skin allergy
Sutton	B	-		-	16,668 $ total hospital costs procedure-related	Stratafix	-	107 (1.8%)
	T				18,759 $ total hospital costs procedure-related	Vicryl, Monocryl, Polysorb, Maxon		190 (3.2%)
Knapper	B	Skin	851	-	-	Quill	-	0
	T		1283			-		1 suture ooze
Thacher	B	Fascia, subcutaneous fat	-	-	-	Quill, Monocryl	-	5 dehiscence, 1 PJI, 2 revisions
	T					Maxon, Monocryl		23 superficial wound infection, 14 purulent drainage, 8 revisions, 3 cellulitis, 3 dehiscence, 2 PJI, 6 abscesses
Serrano Chinchilla	B	Fascia, subcutaneous fat	-	6	-	Quill	-	1 superficial wound infection 1 PJI, 12 suture rupture, 4 hematoma, 2 dehiscence
	T			7		Vicryl		2 superficial wound infection, 1 dehiscence
Wang	B	Fascia, subcutaneous fat	-	Range, 7 to 13	-	Polydioxanon	-	10 fat liquefaction, 9 stich exclusion
Sunderam	B	Fascia		18 ± 21	-	Polydioxanon	1	1 suture abscesses
	T			21 ± 26		Polyglactin	Range, 2 to 4	1 trochanteric bursitis

## Data Availability

Data are available in a separate folder on request.
